# An Integrative Assessment of *Tabanus rubidus* (Diptera: Tabanidae) in Thailand: Distribution, Population Genetics, and Pathogen Surveillance

**DOI:** 10.1155/jotm/8877101

**Published:** 2026-09-11

**Authors:** Kanchana Thinnabut, Wanchai Maleewong, Pairot Pramual, Oranuch Sanpool, Ubon Tangkawanit

**Affiliations:** ^1^ Department of Entomology and Plant Pathology, Faculty of Agriculture, Khon Kaen University, Khon Kaen 40002, Thailand, kku.ac.th; ^2^ Department of Parasitology, Faculty of Medicine, Khon Kaen University, Khon Kaen 40002, Thailand, kku.ac.th; ^3^ Mekong Health Science Research Institute, Khon Kaen University, Khon Kaen 40002, Thailand, kku.ac.th; ^4^ Department of Biology, Faculty of Science, Mahasarakham University, Kantharawichai District, Maha Sarakham 44150, Thailand, msu.ac.th; ^5^ Center of Excellence in Biodiversity Research, Mahasarakham University, Maha Sarakham 44150, Thailand, msu.ac.th

**Keywords:** DNA barcode, environmental factors, genetic variation, haplotype, pathogen

## Abstract

*Tabanus rubidus* (Diptera: Tabanidae) is a significant species of the Tabanidae family, known for its veterinary and medical relevance, and is found across Thailand. This study examines the species distribution, genetic diversity, demographic history, and vector‐pathogen transmission of *T*. *rubidus* collected from all six different regions in Thailand. Molecular techniques, utilizing the *cox*1 gene, were used to assess genetic diversity, genetic structure, and demographic history. The 16S ribosomal RNA gene was used to detect *Anaplasma* and *Ehrlichia*, while the 18S ribosomal RNA gene was used to identify *Trypanosoma* and *Theileria*. *Tabanus rubidus* was found in 23 out of 26 provinces in Thailand, indicating its widespread presence across the country. Negative binomial regression showed that fly abundance was negatively affected by monthly mean temperature but positively influenced by rainfall and site temperature. There was high haplotype diversity and low nucleotide diversity. The median‐joining network network revealed three lineages: Lineage I, which includes Thailand, Malaysia, and Bangladesh; Lineage II, which is found in India and Bangladesh; and Lineage III, which is found in India. Analysis of the demographic history suggested that population expansion started at the end of the Pleistocene. In this study, *T. rubidus* tested negative for *Ehrlichia* and *Theileria*, with *Trypanosoma theileri* (*T. theileri*) and *Anaplasma marginale* being the only detected pathogens. These findings are epidemiologically significant, confirming tabanid‐mediated transmission of *T. theileri* (indicator of trypanosomiasis) and *A. marginale* (agent of bovine anaplasmosis), both of which pose health risks and economic losses to livestock.

## 1. Introduction

Tabanid flies are blood‐feeding insects with over 4665 species in 177 genera [1]. Female *Tabanus* species are of significant medical and veterinary importance due to their hematophagous (blood‐feeding) behavior, which impacts both human and animal populations [2]. Burton [3] reported about 80 different species of horse flies in Thailand. These flies have direct and indirect effects on both animals and humans, serving as mechanical and biological vectors of various pathogens. Raksakoon and Potiwat [4] suggested that the tropical climate of Thailand provides a suitable environment for the proliferation of these vectors, making the region particularly vulnerable to vector‐borne diseases.

*Tabanus rubidus* [5] was first found in West Bengal, India, but there are also populations in Pakistan, the Philippines, China, Indonesia, Thailand, and Myanmar [6, 7]. *T. rubidus* was first identified in Thailand by Burton et al. in 1978, where it is a widely distributed and particularly abundant pest species [8, 9]. This hematophagous species has veterinary and medicinal significance. Its large population size and persistence, particularly in animal farms, contribute to its economic impact and significant role in disease transmission. It has been confirmed as a mechanical vector for pathogens in cattle, such as *Anaplasma marginale* [10], *Bacillus anthracis* [11], and *Trypanosoma evansi* (*T. evansi*) [12, 13]. Additionally, it is also a vector for other pathogens such as *Clostridium chauvoei* and *Pasteurella multocida* [14].

This species is of recognized veterinary significance in the region; however, comprehensive information on its distribution and taxonomic status is still insufficient. Although previous research has employed DNA barcoding with the *cox*1 gene [15] and species identification using the ITS2 sequence [7], these investigations were geographically restricted and do not provide a complete overview of the species across Thailand. Understanding population genetic structure and demographic history through molecular markers can explain the impact of historical environmental changes on current biodiversity. The mitochondrial DNA (mtDNA) *cox*1 sequence was chosen as a genetic marker for studying genetic diversity, genetic structure, and demographic history because it serves as a standard barcoding sequence [16]. With its high mutation rate and maternal inheritance, mtDNA is an essential tool in understanding the evolution and population genetics of invasive species [17]. Its conserved inheritance pattern across various animal species ensures its reliability for evolutionary research [18]. Moreover, studies on pathogens in *T. rubidus* are still limited, and comprehensive data are lacking. Therefore, it is crucial to investigate cattle pathogens for which *T. rubidus* serves as a potential vector. This study focuses on *Anaplasma*, *Ehrlichia*, *Trypanosoma*, and *Theileria*, as these pathogens are responsible for mortality and production losses in cattle [19–21]. For the detection of *Anaplasma* and *Ehrlichia*, which are bacterial pathogens, a typical target gene for molecular identification is the 16S ribosomal RNA (16S rRNA) gene. For the detection of protozoan pathogens, the 18S ribosomal RNA (18S rRNA) gene is used. This gene contains both conserved and variable regions, making it a reliable marker for molecular identification [22].

Given the role of *T. rubidus* as a pathogen vector and the lack of molecular‐based studies, this research aimed to investigate the species distribution, population genetic structure, phylogeography, and demographic history of *T. rubidus* using the mitochondrial cytochrome oxidase I (*cox*1) gene. The study sought to determine the distribution of genetic variation across sampled geographical areas, construct phylogenetic trees to explore the evolutionary relationships among haplotypes, estimate lineage divergence, and identify factors shaping the current phylogeographic pattern of *T. rubidus.* In addition, it can help identify the role of *T. rubidus* as a vector of important cattle pathogens, using 16S rRNA for bacterial detection and 18S rRNA for protozoan parasites. The findings will enhance knowledge of *T. rubidus* in Thailand and contribute to the field of vector epidemiology.

## 2. Materials and Methods

### 2.1. Study Sites

**FIGURE 1:**
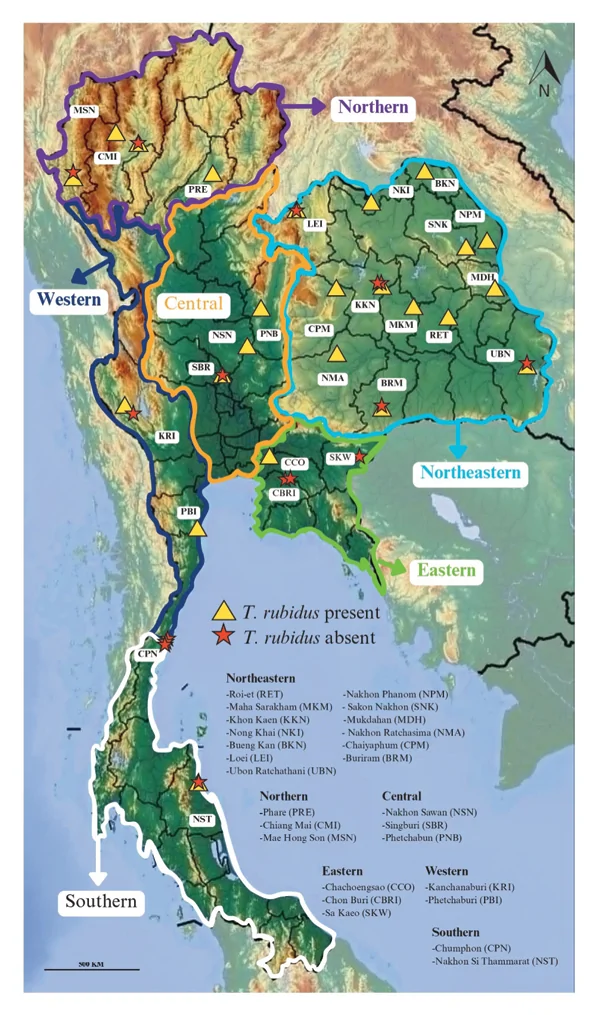
The collection sites of *Tabanus rubidus* specimens span six regions of Thailand: Northern, Southern, Eastern, Western, Northeastern, and Central. The map highlights locations where *T. rubidus* was found (marked with yellow pins) and sites where the species was absent (marked with red pins). Description: Thailand location map Topographic [25]. Source: https://commons.wikimedia.org/wiki/File:Thailand_location_map_Topographic.png. Under a Creative Commons Attribution‐ShareAlike 3.0 Unported (CC BY‐SA 3.0) license.

The study was conducted from January 2024 to November 2024 across 62 beef‐cattle farming areas in Thailand, representing a diverse range of geographical and climatic conditions. The sites were located in 26 provinces and 27 districts, covering all six regions of Thailand: Northern, Southern, Eastern, Western, Northeastern, and Central (Figure 1) (Table A.1). The regional classification was based on geographical, cultural, and climatic variables, as outlined by Kliengchuay et al. [23]. The seasons in Northern, Eastern, Western, Northeastern, and Central regions are three distinct seasons—winter, rainy, and summer—the South only experiences two, such as summer and rainy [24]. Thailand’s location within the tropical monsoon zone and its varied topography influence the climate and weather patterns throughout the country, including the distribution of precipitation. As such, climate differences across the regions were considered important factors for this study. This geographical and climatic variation provided a comprehensive basis for understanding the factors that may influence the distribution of tabanid species in different regions of Thailand.

### 2.2. *Tabanus rubidus* Collection and Morphological Identification

Tabanid specimens, specifically *T. rubidus*, were collected using the Nzi traps [26], which is a standard method for sampling biting flies from beef‐cattle farming areas. Four traps were set up 1 day apart between 1 p.m. and 9 a.m. and checked at 6 p.m. and 9 a.m. in the study area. In addition to the use of Nzi traps, hand‐collection methods were employed in certain areas to ensure comprehensive sampling. After collection, each insect specimen was transferred into 1.5 mL microcentrifuge tubes, and the tubes were immediately placed in a mobile freezer set to −10°C to preserve the specimens until processing. Once in the laboratory, the specimens were stored at −20°C until further analysis. Identification of the collected tabanid specimens was performed under a stereo microscope, following the standard identification keys and guides outlined in Changbunjong et al. [27] and Burton [3].

Climatic data, including temperature, humidity, and the number of cattle, were recorded at each sampling site. Monthly average temperature and rainfall data were also obtained from the Thai Meteorological Department (Table A2) [24].

All sampling procedures adhered to the National Research Council of Thailand’s Guidelines for Animal Experimentation, and ethical approval was obtained from the Animal Ethics Committee of Khon Kaen University (Approval No. IACUC‐KKU‐133/66).

### 2.3. Molecular Study

#### 2.3.1. General Molecular Procedures

Following morphological identification of the specimens, the collected *T* *. rubidus* samples were randomly assigned for DNA extraction. For species identification, one leg of each insect specimen was carefully dissected, while for pathogen detection, the whole body (excluding the wings and legs) was used. DNA extraction was performed using a Macherey‐Nagel DNA Extraction Kit (Macherey‐Nagel GmbH & Co. KG, Germany), following the manufacturer’s instructions. Negative extraction controls were processed alongside the samples during DNA extraction to monitor possible contamination. The extracted DNA was quantified using a NanoDrop spectrophotometer. The DNA concentration ranged from 0.5 to 505.6 ng/μL. The DNA template concentration was standardized based on the TOYOBO (Japan) PCR protocol, which recommends an input of 10 ng per 50 μL reaction. In this experiment, 25 μL was used per reaction. The amount of DNA template was adjusted to achieve 5–100 ng of DNA input in a single 25 μl PCR, according to the manufacturer’s protocol (Quick Taq HS DyeMix 2x; TOYOBO, Japan). A no DNA template control (negative control) was included in every PCR run to monitor contamination, and no amplification was detected in any of the negative controls. To minimize cross‐contamination, DNA extraction, PCR setup, and post‐PCR analyses were conducted in physically separated laboratory areas. Representative PCR products were visualized using agarose gel electrophoresis to verify successful amplification and were delivered to sequencing services (1st BASE or Apical Scientific Sdn. Bhd.) in Selangor, Malaysia, for nucleotide sequencing.

#### 2.3.2. Molecular Identification and DNA Amplification

From each province, some of the *T. rubidus* specimens (*n* = 47 in total) were randomly selected for species identification using molecular techniques. To amplify a section of the *cox1* gene (∼658 bp), the forward and reverse primers LepF1 (5′‐ATTCAACCAATCATAAAGATATTGG‐3′) and LepR1 (5′‐TAAACTTCTGGATGTCCAAAAAATCA‐3′) were used. The *cox*1 primers used in this study were based on those described by Hebert et al. [28]. Each 25 μL reaction included the following: 0.5 μL of each primer, 2 μL of template DNA, 12.5 μL of Quick Taq Master Mix, and 9.5 μL of deionized water. The PCR conditions were as follows: initial denaturation at 95°C for 5 min, followed by 35 cycles of 94°C for 35 s, 45°C for 45 s, 72°C for 1 min, a final extension at 72°C for 7 min, and cooling at 4°C.

#### 2.3.3. Pathogen Detection

For pathogen detection of *Anaplasma*, *Ehrlichia*, *Theileria*, and *Trypanosoma*, 100 (49.75%) *T. rubidus* samples were randomly collected. Positive controls consisting of reference DNA from previously confirmed pathogen samples were included in each PCR run to verify amplification efficiency. Positive controls for *Anaplasma*, *Ehrlichia*, and *Theileria* were obtained from previous studies [29, 30], while the *Trypanosoma*‐positive control was obtained from the Mekong Health Science Research Institute. The 16S rRNA gene was amplified for *Anaplasma* and *Ehrlichia* using primers EHR1F (5′‐GAACGAACGCTGGCGGCAAGC‐3′) and EHR2R (5′‐AGTAYCGRACCAGATAGCCGC‐3′) [31], producing a 686 bp product. For *Theileria,* the 18S rRNA gene was amplified using primers Piro1‐S (5′‐CTTGACGGTAGGGTATTGGC‐3′) and Piro3‐AS (5′‐CCTTCCTTTAAGTGATAAGGTTCAC‐3′) [32], yielding fragments of 1407–1409 bp. For *Trypanosoma*, the 18S rRNA gene was amplified using primers 18SrRNAF3 (5′‐ATGTCATGCATGCGAGGGGGCGTCCGTG‐3′) and 18SrRNAR3 (5′‐CACCTCTGACGCACCAGTACGTTCTCCCCC‐3′), resulting in a 368 bp product. All detection protocols used 25 μL reactions containing 0.5 μL of each primer (10 μM), 4 μL of template DNA, 12.5 μL of Quick Taq Master Mix, and 7.5 μL of deionized water. Thermal conditions were 94°C for 5 min, followed by 35 cycles of denaturation at 94°C for 30 s, primer annealing at 60°C (*Anaplasma* and *Ehrlichia*), 48.5°C (*Theileria*), and 62°C (*Trypanosoma*) for 30 s, and extension at 72°C for 45 s, with a final extension at 72°C for 7 min and cooling at 4°C.

### 2.4. Data Analysis

#### 2.4.1. Genetic Diversity and Demographic History of *Tabanus rubidus*

The sequences of *T. rubidus* were aligned using the BioEdit Sequence Alignment Editor software [33]. These sequences were then compared with those available in the public database, GenBank, to assess their genetic relationships (Table A3). To estimate genetic diversity, haplotype diversity (*h*) and nucleotide diversity (*π*) were calculated using the K2P model (Kimura 2‐parameter model) in Arlequin Version 3.5.2.2 [34]. Populations were grouped using DnaSP6 [35] with 1000 permutations performed using Arlequin 3.5.2.2 [34].

The median‐joining (MJ) network approach [36] was used to evaluate 107 *T* *. rubidus* sequences, including 47 newly obtained sequences and an additional 60 sequences retrieved from GenBank and BOLD systems (Table A.4). The haplotype network was generated using PopART software Version 1.7 [37]. To assess the demographic history of *T. rubidus*, the mismatch distribution was used. According to Rogers and Harpending [38], a population that has recently undergone demographic expansion is expected to exhibit a unimodal mismatch distribution. The divergence from the predicted sudden expansion model was tested using the sum‐of‐squares deviation (SSD) and Harpending’s raggedness index [39]. Additionally, Tajima’s D [40] and Fu’s F_S_ [41] statistical tests were applied to evaluate whether the population was in equilibrium.

The expansion time of the population was estimated using the formula *τ* = 2 ut, where *t* is the generation time, *u* = *m*_T_ *μ* (*μ* is the mutation rate per nucleotide and *m*_T_ is the length of the nucleotide sequences) (624 bp) [38]. For this study, *T. rubidus* was assumed to produce 3.5 generations annually [42].

#### 2.4.2. Analysis of Climatic Factors and Cattle Density

The abundance of female *T. rubidus* was analyzed using negative binomial (NB) regression to address the inherent overdispersion in the count data. The model was constructed to evaluate the influence of five environmental predictors: site temperature, monthly mean temperature, rainfall, livestock count, and mean humidity. To ensure the reliability of the findings, model selection was performed based on the Akaike information criterion (AIC), where the model with the lowest AIC value was selected as the most parsimonious. The presence of multicollinearity was assessed using the variance inflation factor (VIF), ensuring no significant redundancy between predictors. The dispersion parameter (*θ*) was monitored to verify that the NB framework appropriately corrected for the data’s variance‐to‐mean relationship. All analyses were conducted in R using the MASS package.

#### 2.4.3. Pathogen Detection in *Tabanus rubidus*

The sequences were aligned using the BioEdit Sequence Alignment Editor software [33]. These sequences were then compared with those available in the public database, GenBank, to assess their genetic relationships. The sequence dataset of *Trypanosoma* and *Anaplasma* was used to construct phylogenetic trees using the maximum‐likelihood (ML) and neighbor‐joining (NJ) methods under the Kimura 2‐parameter model with 1000 bootstrap replicates in MEGA 12 [43].

## 3. Results

### 3.1. Distribution and Genetic Diversity of *T. rubidus* in Thailand

A total of 201 adult female *T. rubidus* specimens were collected from 62 beef‐cattle farming areas across 26 provinces and 27 districts, covering all six regions of Thailand. The study confirmed the presence of *T. rubidus* in 23 provinces across all regions. There were only three locations, from Chumphon in the south and Chon Buri and Sa Kaeo in the east, where this species was not found (Table A.1). The specimens were identified based on their morphological characteristics and supported by *cox*1 gene sequencing.

A total of 47 *cox*1 gene sequences (624 bp) from *T. rubidus* specimens collected in Thailand revealed 37 unique haplotypes. Five of these haplotypes were shared across multiple provinces, including haplotypes H3 (PBI18, CMI30), H17 (PRE12, SBR3), and H20 (PBI8, CPM33). Haplotype H11 was found in four provinces (NSN8, PNB38, NPM6, NMA6), while haplotype H8 was present in five provinces (CMI24, RET1, MDH1, CCO22, CPM31). The remaining 32 haplotypes were unique to particular regions. Sequence comparison with GenBank data revealed 98.40%–100% similarity to *T. rubidus*. The 36 haplotypes from five regions (Northern, Eastern, Western, Northeastern, and Central) were similar to *T. rubidus* previously reported in Thailand. However, one haplotype (H25: NST8) from the Southern region showed 99.84% similarity to *T. rubidus* from Malaysia (Table A.3).

Analysis of the *cox*1 gene across Thailand revealed high haplotype diversity within each location. For provinces that had more than one haplotype, a detailed analysis of haplotype and nucleotide diversity was performed (Table 1). Despite the high haplotype diversity, nucleotide diversity was low, especially in Ubon Ratchathani (UBN) Province (Table 2). The average nucleotide diversity of all haplotypes was 0.0086 ± 0.0047, with a haplotype diversity of 0.9824 ± 0.0104.

**TABLE 1:** Negative binomial regression analysis of factors affecting female *Tabanus rubidus* abundance.

Variable	Coefficients (*β*)	Std. error	*p*‐value
(Intercept)	17.567	4.398	< 0.001^∗∗^
Site Temperature	0.107	0.040	0.007^∗^
Monthly mean temperature	−20.658	0.155	< 0.001^∗∗^
Rainfall (mm)	0.007	0.002	0.008^∗^
Livestock count	0.008	0.024	0.733^ns^
Mean humidity	−0.015	0.018	0.407^ns^

*Note:* Temperature was measured in the field during sampling as the afternoon time. Monthly mean temperature, humidity, and rainfall data were obtained from the Thai Meteorological Department [24].

Abbreviation: ns = not significant.

^∗^ *p* < 0.05.

^∗∗^ *p* < 0.001.

**TABLE 2:** Haplotype and nucleotide diversity of the *cox*1 gene in *Tabanus rubidus* from Thai provinces with more than one haplotype.

Province	No. of haplotypes	Haplotype diversity (*h*)	Nucleotide diversity (*π*)	Segregating sites (*S*)	Nucleotide differences (*k*)
CMI	8	1.0000 ± 0.0625	0.0101 ± 0.0061	20	6.2857 ± 3.3374
MKM	3	1.0000 ± 0.2722	0.0085 ± 0.0071	8	5.3333 ± 3.5277
PBI	2	1.0000 ± 0.5000	0.0144 ± 0.0152	9	9.0000 ± 6.7082
CPM	2	1.0000 ± 0.5000	0.0112 ± 0.0120	7	7.0000 ± 5.2915
PNB	3	1.0000 ± 0.2722	0.0096 ± 0.0079	9	6.0000 ± 3.9279
UBN	2	1.0000 ± 0.5000	0.0048 ± 0.0056	3	3.0000 ± 2.4495
BRM	2	1.0000 ± 0.5000	0.0096 ± 0.0104	6	6.0000 ± 4.5826
MSN	2	1.0000 ± 0.5000	0.0144 ± 0.0152	9	9.0000 ± 6.7082
SNK	2	1.0000 ± 0.5000	0.0160 ± 0.0168	10	10.000 ± 7.4162
CCO	2	1.0000 ± 0.5000	0.0128 ± 0.0136	8	8.0000 ± 6.0000
NKI	2	1.0000 ± 0.5000	0.0176 ± 0.0184	11	11.0000 ± 8.1240
SBR	3	1.0000 ± 0.2722	0.0085 ± 0.0071	8	5.3333 ± 3.5277
LEI	3	1.0000 ± 0.2722	0.0064 ± 0.0054	6	4.0000 ± 2.7255
Total^∗^	37	0.9824 ± 0.0104	0.0086 ± 0.0047	—	—

^∗^Total haplotypes identified across all study areas in Thailand.

The nucleotide diversity (*π*) exhibited moderate variation among the populations, with an overall mean of 0.0086 ± 0.0047. The highest nucleotide diversity was observed in Nong Khai (NKI) (*π* = 0.0176 ± 0.0184), while the lowest was recorded in UBN (*π* = 0.0048 ± 0.0056) (Table 2).

The number of segregating sites (*S*) ranged significantly from a minimum of 3 in UBN to a maximum of 20 in Chiang Mai (CMI). Correspondingly, the average number of nucleotide differences (*k*) followed a similar trend, peaking in NKI (11.0000 ± 8.1240) and remaining lowest in UBN (3.0000 ± 2.4495). These indices collectively suggest a highly polymorphic population structure with distinct genetic signatures across the sampled provinces (Table 2).

### 3.2. Environmental Factors Associated With *Tabanus rubidus* Abundance

The environmental factors influencing the abundance of *T. rubidus* were evaluated using an NB regression model. The model showed a high level of goodness of fit with an AIC of 275.98 and a dispersion parameter (*θ*) of 0.748, indicating its effectiveness in handling the overdispersed nature of the insect count data. The results demonstrated that monthly mean temperature had a highly significant negative impact on fly abundance (*β* = −0.658, *p* < 0.001), suggesting that higher sustained temperatures throughout the month lead to a decrease in the population. Conversely, rainfall was found to have a significant positive correlation with the number of flies (*β* = 0.004, *p* = 0.008), indicating that increased precipitation supports higher fly density. Furthermore, the temperature at the time of sampling significantly and positively influenced the observed fly counts (*β* = 0.107, *p* = 0.007), likely due to increased flight activity during warmer periods. However, other factors, including livestock count and mean humidity, did not show statistically significant associations with fly abundance in this model (*p* > 0.05).

### 3.3. Demographic History of *Tabanus rubidus* Populations

**FIGURE 2:**
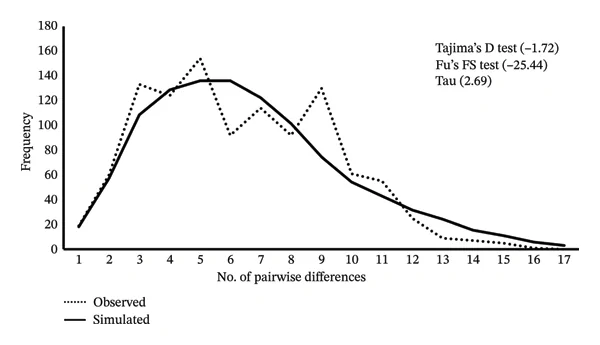
Mismatch distribution pattern of *Tabanus rubidus cox*1 sequences in Thailand is consistent with the predictions of the sudden population expansion model (SSD = 0.0063, *p* > 0.0500; Harpending’s raggedness index = 0.0173, *p* > 0.0500).

The demographic history of *T. rubidus* populations was examined by analyzing deviations from neutrality and changes in population size. A recent population expansion is defined by a unimodal mismatch graph, as shown in Figure 2 using mismatch distribution analysis. SSD (SSD = 0.00621139, *p* = 0.25) and Harpending’s raggedness index (0.01730761, *p* = 0.15) were not significantly different from the simulated data predicted under the sudden population expansion model (Figure 2). Using a *cox*1 divergence rate of 0.0354 per site per million years [44] and assuming 3.5 generations per year, the population expansion was estimated to have occurred approximately 40,952 years ago.

### 3.4. Mitochondrial Genealogy

**FIGURE 3:**
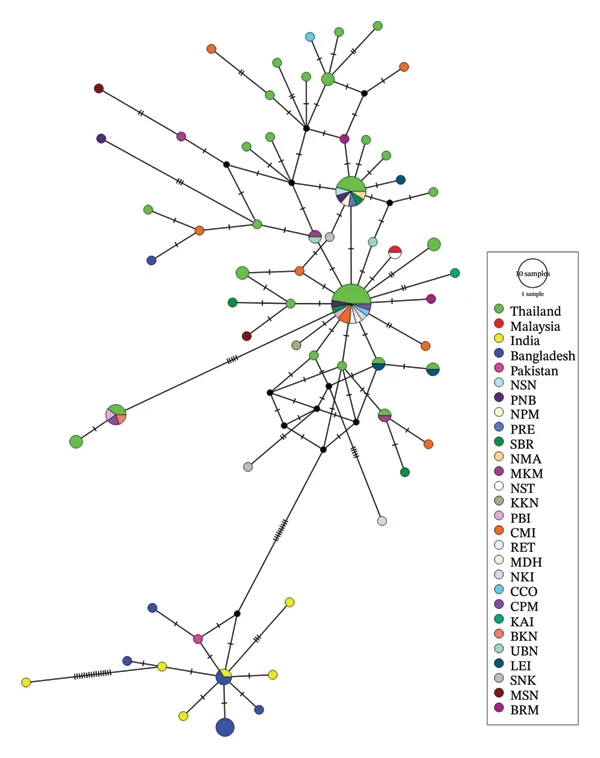
The median‐joining network of 107 *cox*1 sequences (47 from this study and 60 from other studies) of *Tabanus rubidus* is presented. Each circle in the network represents a haplotype, with the size of the circle corresponding to the number of individuals possessing that specific haplotype. The haplotypes are labeled based on their province and country of origin. The number of slashes across the lines connecting the haplotypes reflects the number of mutations inferred to have occurred along that particular branch. Median vectors are represented by small black circles.

The MJ network was constructed using 107 *cox*1 sequences, each with a length of 546 bp after trimming. These included 47 sequences from this study and 60 additional sequences from other sources, as shown in Table A.4. The MJ network displayed a star‐shape, with Haplotype 8 from this study serving as a central haplotype, shared by widely dispersed *T. rubidus* populations across Thailand. The MJ network related and formed a distinct lineage (Lineage I) including Thailand, Malaysia, and Bangladesh; Lineage II was more distantly related to the sequences from Bangladesh and India, whereas Lineage III was formed by one sequence from India. Lineage II was linked to Lineage I by eight mutation steps, while Lineage III was linked to Lineage I by 15 steps (Figure 3). The genetic distance between Lineage I and Lineage II was 2.42%, between Lineage I and Lineage III was 3.51%, and between Lineage II and Lineage III was 3.39%.

A total of 61 haplotypes were identified across the studied regions, exhibiting distinct geographic distribution patterns. The distribution can be categorized into widespread haplotypes and those with localized or restricted distributions. The Thai populations were characterized by two predominant and widely dispersed lineages. Haplotype 19, the most prevalent lineage, was distributed extensively across nine provinces, including Phetchaburi, CMI, Roi Et, Mukdahan, NKI, Chachoengsao, Chumphon, Phetchabun, and Sing Buri. Haplotype 1 showed broad distribution across Nakhon Sawan, Phetchabun, Nakhon Phanom, Phrae, Sing Buri, and Nakhon Ratchasima. Significant regional genetic differentiation was observed through numerous localized haplotypes. Specific lineages were restricted to individual provinces, such as CMI (Hap 20–23, 25–26), Loei (Hap 32–33, 43), and Mae Hong Son (Hap 31, 36). Haplotypes 50–61 were exclusively identified within Thailand.

### 3.5. Pathogen Detection in *Tabanus rubidus*

**FIGURE 4:**
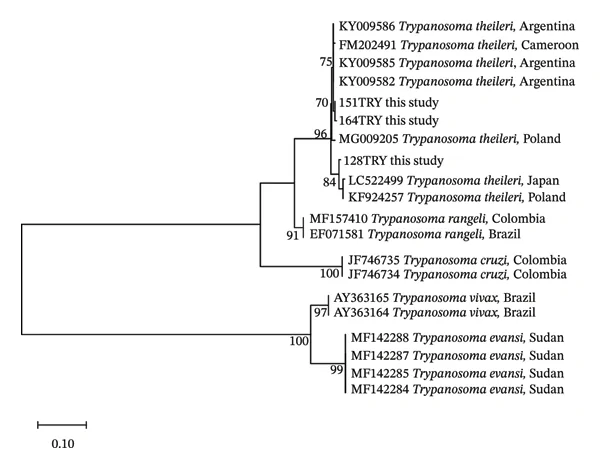
Maximum‐likelihood tree of *Trypanosoma* species inferred from 18S rRNA gene 17 sequences retrieved from NCBI GenBank plus 3 collected from *Tabanus rubidus* in Thailand.

A total of 100 (49.75%) *T. rubidus* specimens from 23 provinces in Thailand were screened for *Anaplasma, Ehrlichia, Theileria,* and *Trypanosoma*. The results showed no detection of *Ehrlichia* and *Theileria.* Molecular detection revealed *Trypanosoma* in 5 out of 100 specimens (5%). The five *Trypanosoma* specimens were identified as *T. theileri* (99.07%–99.69% similarity) originating from Loei (*n* = 2), Phrae (*n* = 1), Mae Hong Son (*n* = 1), and Kanchanaburi (*n* = 1). Furthermore, three specimens (3%) were positive for *A*. *marginale* (100% similarity), distributed in Loei (*n* = 2) and Maha Sarakham (*n* = 1). The ML tree (Figure 4) based on the three 18S rRNA sequences of *Trypanosoma* obtained from *T. rubidus* in livestock farm areas in Thailand plus 20 of the most closely related sequences retrieved from NCBI GenBank revealed two major clades. Clade I consisted of 3 species: *T. theileri*, *T. rangeli*, *and T. cruzi.* 151TRY (PV998199) and 164TRY (PV998200) were included in the same clade as *Trypanosoma theileri* from Cameroon and Argentina, with a bootstrap value of 75% for the ML tree, and closely related to Poland, with a bootstrap value of 70%. Additionally, 128TRY (PV998198) was included in the same subclade as *Trypanosoma theileri* from Japan and Poland with a bootstrap value of 84%. Clade II was divided into two subclades. Subclade I consisted of *T*. *evansi* from Sudan, and Subclade II was composed of *T. vivax* from Brazil.

**FIGURE 5:**
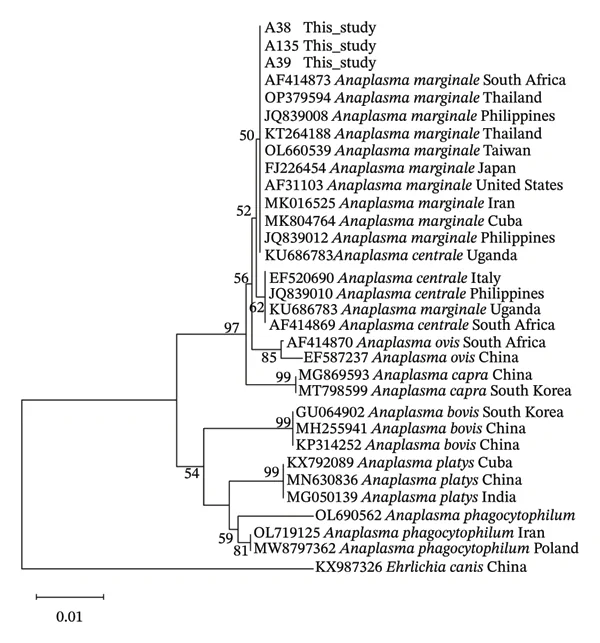
Neighbor‐joining tree of *Anaplasma* species inferred from 28 sequences of the 16S rRNA gene retrieved from NCBI GenBank plus 3 sequences obtained from *Tabanus rubidus* in Thailand.

The NJ tree based on 642 bp 16S rRNA sequences (Figure 5) illustrated the phylogenetic relationship of the three *Anaplasma* isolates obtained from *T. rubidus* in this study (GenBank accession numbers of A38 [PZ437203], A39 [PZ437202], and A135 [PZ437201]), compared with 11 closely related sequences retrieved from NCBI GenBank. The specimens from this study were grouped with *A. marginale* strains isolated from South Africa, Thailand, the Philippines, Taiwan, Japan, the United States, Iran, Cuba, and Uganda.

## 4. Discussion

In this study, *T. rubidus* was found in 23 of 26 provinces surveyed in Thailand, indicating that it was widespread in the country. These findings corresponded with the results of Changbunjong et al. [8], who identified *T. rubidus* along with *T. megalops* and *T. striatus*, and as the commonest species. Changbunjong et al. [8] reported that *T. rubidus* was more frequently found in villages than in primary and secondary forests, indicating a potential association with human settlements or activities. This corresponds to the sites where we found the samples.

The high level of polymorphism observed, particularly in the CMI population with 20 segregating sites, suggests that these populations maintain a significant reservoir of genetic variation. This is further highlighted by the highest nucleotide diversity found in NKI (*π* = 0.0176). High genetic diversity in vector populations is often associated with a greater capacity for adaptation to local environments, including the development of insecticide resistance and varying efficiencies in disease transmission. Furthermore, the decrease in the total haplotype diversity (*h* = 0.9824) compared to the maximum diversity observed in individual provinces (*h* = 1.0000) provides strong evidence of shared haplotypes across different geographic regions.

*T. rubidus* was found in all six regions across Thailand, but the abundance of individuals at each location was different (Table A.1). The results showed that *T. rubidus* was more abundant in provinces in the Northern and Northeastern regions than in provinces in other regions. Several factors may explain this observation. The study was conducted from January 2024 to November 2024 across 62 beef‐cattle farming sites in Thailand. The environmental data revealed a wide range of climatic conditions that likely influence the population dynamics of *T. rubidus*. The recording of sampling‐period temperatures as high as 44.1°C combined with varying humidity levels suggests that these vector populations possess high physiological plasticity. Furthermore, the substantial monthly rainfall recorded in certain provinces (up to 404.6 mm) provides optimal conditions for larval habitat expansion, potentially explaining the high haplotype diversity observed in this study.

Beyond abiotic influences, interspecific competition may serve as a key biotic factor explaining the observed variations in population density. In certain sampling sites where a high diversity of other fly species was recorded, the abundance of *T. rubidus* was notably lower. This observation aligns with the hypotheses proposed by de Oliveira Zamarchi et al. [45], suggesting that tabanid abundance can be constrained by both environmental factors and competition as a mechanism for ecological niche partitioning.

However, the specific role of interspecific competition within the diverse agricultural landscapes of Thailand remains a hypothesis that requires further empirical validation in future studies. Subsequent research should incorporate broader species diversity assessments and more detailed biological interaction analyses to overcome the limitations of short‐term sampling. As noted, collecting samples for only 1 day per site across different seasons may influence the observed distribution and the representativeness of each locality. Addressing these biotic interactions will provide a more comprehensive understanding of population dynamics than physical climatic parameters alone.

Our regression analysis underscores the complex, two‐faceted role of ambient temperature in regulating *T. rubidus* population dynamics. Most notably, monthly mean temperature exerted a highly significant negative impact on fly abundance (*β* = −0.658, *p* < 0.001), suggesting that sustained, elevated temperatures over an extended period act as a macroclimatic constraint. This detrimental effect is likely attributed to the thermal limits of tabanid immatures, where prolonged high temperatures may accelerate soil desiccation, thereby reducing the survival rates of larvae and pupae residing in moist topsoil. Conversely, the immediate site temperature demonstrated a significant positive correlation with observed fly counts (*β* = 0.107, *p* = 0.007). This apparent paradox can be biologically explained by the microclimatic stimulation of adult behavior; warmer ambient temperatures during sampling hours act as an immediate physiological trigger that enhances flight activity and host‐seeking behavior in adult females. Specifically, an increase in temperature at the collection sites directly drives elevated flight intent and foraging intensity among female flies. This behavioral surge is strongly consistent with previous investigations by Baldacchino et al. [46] and Thinnabut et al. [47], who documented that higher microclimatic warmth acts as a primary catalyst for intensified host‐seeking patterns in tabanids. Consequently, while long‐term heat suppresses overall population recruitment, short‐term warmth promotes immediate vector activity.

Furthermore, the significant positive association with rainfall (*β* = 0.004, *p* = 0.008) reinforces the critical dependence of *T. rubidus* on environmental moisture. Increased precipitation directly expands and maintains semiaquatic or mud‐rich breeding microhabitats, which are vital for larval development. In contrast, the lack of significant association with mean humidity and livestock count (*p* > 0.05) indicates that these variables may not function as immediate limiting factors within the studied ecotype, or their influences might be modulated through indirect ecological pathways. From a statistical perspective, the robust fit of the NB model (AIC = 275.98, *θ* = 0.748) confirms its mathematical validity in accommodating the overdispersed nature of insect count data, validating the reliability of these ecological predictors. Ultimately, understanding these climate‐driven population shifts is crucial, as the synchronization of high rainfall and optimal daytime temperatures creates ideal windows for tabanid proliferation, exponentially increasing the risk of mechanical transmission of pathogens such as *Anaplasma marginale* within Thai livestock herds.

Haplotype analysis of *T. rubidus* using the *cox*1 gene revealed 37 haplotypes from 47 specimens. The findings indicate a high level of haplotype diversity but low nucleotide diversity, indicating a prevalence of closely related haplotypes. This observation suggests that the population might have undergone a recent expansion [48]. According to Katsares et al. [49], high haplotype diversity generally indicates significant gene flow between populations, whereas low nucleotide diversity indicates low genetic variation between haplotypes, possibly due to population expansion following a period of low effective population size [50]. This supports the findings of Tajima’s D and Fu’s F_S_ tests, as well as the mismatch distribution analysis, which suggested a recent population expansion in this species. Similar patterns of genetic diversity have been observed in other insect species in Thailand, such as *Anopheles minimus* [51] and *Culicoides mahasarakhamense* [52]. This could be due to these two areas being the center of population expansion. CMI province has the highest haplotype diversity (8 haplotypes, from 3 locations; Table 2). One possible reason for high diversity is population size. Hague and Routman [53] revealed that genetic diversity will increase with a larger effective population size and decreasing effects of drift. Haplotype sharing with populations in the North, Northeast, and Central regions (Haplotypes 3, 8, 11, 17, and 20), which are genetically closely related to the most common haplotype, indicates that there has been gene flow among populations in these regions. Haplotype diversity found in this study may be related to the morphological variation of mosquito species revealed by Chaiphongpachara et al. [54]. They found intraspecific variation in wing geometry among *T. rubidus* populations from four different geographical regions in Thailand.

In this study, an MJ network based on the *cox*1 gene sequence was used to explore the relationships between *T. rubidus* populations from Thailand, India, Bangladesh, Pakistan, and Malaysia, where the *cox*1 gene sequence is available. The presence of numerous unique haplotypes across all populations agrees with the detection of recent population demographic expansion. The results indicate that one sample from Bangladesh is placed in the same lineage as those from Thailand and Malaysia. This suggests the hypothesis that (1) Bangladesh and Thailand share certain ecological features, such as tropical and subtropical climates. This could allow for the migration or spread of species across these areas, where similar habitats and resources are available for the insect to survive, and (2) haplotypes from Bangladesh simply exist in Thailand due to genetic drift or migration, even though the populations are geographically distinct. *T. rubidus* is a geographically widespread species that is distributed in Asia [6, 7], allowing for genetic drift and gene flow, even over long distances. The study also revealed that *T. rubidus* specimens from Nakhon Si Thammarat are the same haplotype as one sequence that was only reported in Malaysia (MZ769389). This could be related to the similar ecological conditions in Malaysia and southern Thailand. Parts of southern Thailand, such as Nakhon Si Thammarat, were part of the Thai–Malay Peninsula, which, at that time, was connected to the Indonesian archipelago by land [55]. Insects in Thailand, such as head lice, have been reported sharing similar haplotypes with those found in Malaysia [56]. Although three lineages were observed in the MJ network, this pattern should be interpreted cautiously. Alternative explanations such as sampling bias, incomplete lineage sorting, and limited taxon coverage cannot be excluded, and the data do not allow strong inference of historical biogeographic separation.

The recent history as indicated by population demographic expansion dating back to only approximately 40,952 years ago may explain the observed genetic patterns. In this study, the sample size for the population genetic analysis was limited due to the availability of specimens from certain regions. Although smaller sample sizes may reduce the statistical power to detect subtle genetic patterns, the results still provide valuable insights into the genetic structure of the populations studied. Nevertheless, the findings should be interpreted with caution.

In Thailand, reports of pathogen findings in tabanids have been reported. For example, *Anaplasma*, *Ehrlichia*, and *Theileria* were found in *T. megalops* [57], and Gomontean et al. [58] reported the presence of the *Trypanosoma theileri* complex in *Chrysops dispar*. Additionally, *Tabanus* species have been linked to *Theileria orientalis* and *T. sinensis* infections [59]. However, this study did not detect *Ehrlichia* or *Theileria* in *T. rubidus*. The relatively low prevalence of these pathogens in our study could be closely linked to the nature of their transmission mechanisms and sampling dynamics. Unlike biological vectors, where pathogens replicate and persist within the host for life, tabanids predominantly facilitate mechanical transmission, meaning pathogen survival on their mouthparts is temporary and highly dependent on active infections in local herds during the sampling window. Furthermore, this low detection or absence in the majority of samples could result from additional factors, such as low initial pathogen levels in the vector, minor sample contamination, or the rapid degradation of pathogens postcollection [46, 60–62]. Consequently, our findings capture a realistic, baseline snapshot of mechanical transmission dynamics in the area.

However, the study identified five pathogen‐positive specimens with 99.07%–99.69% similarity to *Trypanosoma theileri* through NCBI BLAST analysis. This represents the first report of this pathogen in *T*. *rubidus* in Thailand. This finding is epidemiologically relevant because *T. theileri* can serve as an indicator of tabanid‐mediated trypanosome transmission in cattle populations. Although this species is generally considered nonpathogenic or only mildly pathogenic, it can still lead to clinical effects under certain conditions. In cattle that are heavily infected or immunocompromised, *T. theileri* infection may cause mild anemia, weight loss, reduced milk yield, diarrhea, emaciation, and decreased productivity. In more serious instances, it can cause mortality in fetuses and newborn calves [63–66]. Moreover, its presence highlights active contact between cattle and biting flies, reflecting the potential risk of mechanical transmission of other, more pathogenic trypanosomes such as *T. evansi* [13, 46, 67]. Therefore, detecting *T. theileri* in *T. rubidus* suggests not only its circulation in Thai cattle populations but also the existence of ecological conditions favorable for trypanosome transmission, underscoring the importance of continued surveillance and vector control in livestock management.

The ability of *T. theileri* to be transmitted through both mechanical and biological routes [68] increases its chances of being detected. Tabanid feeding behavior is an important factor in mechanical transmission; when a tabanid carrying the parasite is disturbed during feeding, it repeatedly moves to new hosts until fully engorged. As a result, a single infected tabanid can transmit the pathogen multiple times within one feeding period.

ML analysis of 18S rRNA sequences showed that *Trypanosoma* sequences (128TRY, 151TRY, and 164TRY) from *T. rubidus* in Thailand clustered with *T. theileri* reference sequences from GenBank (Figure 4). These sequences were obtained from Loei, Phrae, and Kanchanaburi provinces, indicating that *T. theileri* is present across multiple regions of Thailand and may be broadly distributed in the country’s ruminant population.

A major finding of this study is the identification of *A*. *marginale* within *Tabanus rubidus* specimens via 16S rRNA gene screening. This represents the first report of this pathogen in *T. rubidus* in Thailand. Notably, these infected specimens were recovered from Loei and Maha Sarakham provinces. This geographical discovery possesses profound epidemiological relevance, given that *A. marginale* drives bovine anaplasmosis—a debilitating and financially ruinous disease in cattle. Clinically, affected livestock typically manifest severe hemolytic anemia, marked lethargy, icterus, and anorexia. In acute, high‐grade infections, the massive destruction of parasitized erythrocytes induces systemic hypoxia, ultimately culminating in mortality [69]. Suppressing this pathogen remains highly challenging, as a universally protective and safe vaccine against bovine anaplasmosis is currently unavailable [70]. Consequently, livestock producers must rely extensively on antimicrobials and vector management. Against this backdrop, the detection of *A. marginale* DNA in *T. rubidus* highlights the important role of these blood‐feeding flies in disease transmission. Furthermore, our findings suggest that Loei and Maha Sarakham provinces should be prioritized for rigorous surveillance programs and stringent preventive measures to curb potential outbreaks.

Driven by the interrupted feeding patterns inherent to tabanids—wherein the fly is frequently disturbed by the host’s defensive actions and repeatedly seeks adjacent hosts to satisfy its blood meal—*T. rubidus* functions as an exceptionally potent mechanical vector. This particular feeding strategy permits a single infective fly to inoculate multiple susceptible hosts within a brief period, thereby triggering rapid outbreaks across herds. This phenomenon highlights the imperative to prioritize the suppression of biting fly populations as a cornerstone preventive measure.

Furthermore, the biothreat posed by *T. rubidus* extends into the realm of human health. Under high population densities, these flies display intensely aggressive biting habits and frequently attack livestock handlers and farmers. This opportunistic feeding on humans not only inflicts painful lesions and localized dermal irritation but also introduces a potential, though frequently disregarded, risk of mechanical transmission of zoonotic or livestock pathogens to humans. Accordingly, our insights indicate that managing *T. rubidus* populations transcends veterinary medicine; it represents a crucial public health and biosecurity necessity requiring sustained monitoring and integrated vector mitigation strategies.

Our phylogenetic analysis using the NJ method (Figure 5) further supports these findings, as the *Anaplasma* sequences (A38, A39, and A135) derived from *T. rubidus* clustered tightly with *A. marginale* reference strains from diverse global origins. This study revealed that *T. rubidus* is a species with no recognized sibling species, as supported by current morphological and molecular evidence, and is widely distributed across cattle farms in Thailand. Observations revealed high haplotype diversity but low nucleotide diversity, which conforms to the hypothesis of a recent population expansion. Also, low genetic differentiation between populations could be a result of this historical factor. In addition, the high dispersal ability could facilitate gene flow and, therefore, reduce population genetic differentiation. Furthermore, *T. rubidus* is the vector pathogen of *T. theileri* and *A. marginale* in this study area, playing a key role in trypanosome transmission and causing clinical effects that ultimately impact animal health and productivity.

## Author Contributions

Kanchana Thinnabut: data curation, formal analysis, investigation, methodology, writing–original draft, and writing–review and editing. Wanchai Maleewong: conceptualization, methodology, supervision, visualization, and writing–review and editing. Pairot Pramual: supervision and writing–review and editing. Oranuch Sanpool: resources and validation. Ubon Tangkawanit: conceptualization, funding acquisition, methodology, project administration, supervision, writing–original draft, and writing–review and editing.

## Funding

This research was funded by the National Research Council of Thailand (NRCT) (Contact No. N41A661127) and the Research Department of Khon Kaen University. This project was partially funded by the grant from the National Research Council of Thailand (NRCT): High‐Potential Research Team Grant Program (Contract no. N42A670561 to Wanchai Maleewong).

## Conflicts of Interest

The authors declare no conflicts of interest.

## Supporting Information

Additional supporting information can be found online in the Supporting Information section.

## Supporting information


**Supporting Information** Supporting Table A.1 *Tabanus rubidus* specimens were collected from beef‐cattle farms across Thailand between January 2024 and November 2024. The sampling took place at various sites located in the six geographical regions of Thailand: Northern, Southern, Eastern, Western, Northeastern, and Central regions. Supporting Table A.2 Climatic conditions at sampling sites during the collection period, including temperature, humidity, and monthly average rainfall by province. Supporting Table A.3 Haplotype diversity (Hd) and percentage sequence similarity of *Tabanus rubidus* COI sequences (624 bp) from beef‐cattle farming areas in Thailand, compared with reference sequences from GenBank. Supporting Table A.4 List of Tabanidae COI sequences used for median‐joining network analysis, showing geographical origins and database identifiers.

## Data Availability

The data that support the findings of this study are available on request from the corresponding author. The data are not publicly available due to privacy or ethical restrictions.
