# Supplementary material for: An Integrative Assessment of Tabanus rubidus (Diptera: Tabanidae) in Thailand: Distribution, Population Genetics, and Pathogen Surveillance
Source: J Trop Med. 2026 Sep 11;2026:8877101. doi: 10.1155/jotm/8877101 (PMC13563500; doi:10.1155/jotm/8877101)
Supplement: Supplementary file 1 [file JOTM-2026-8877101-s001.docx]

**Table A.1** *Tabanus rubidus* specimens were collected from beef-cattle farms across Thailand between January 2024 and November 2024. The sampling took place at various sites located in the six geographical regions of Thailand: Northern, Southern, Eastern, Western, Northeastern, and Central regions.

|  | **Province** | **District** | **Date** | **No. Farm** | **GPS** | **Elevation (M)** | **No** | **No.** | **No.** | **No.** |
| --- | --- | --- | --- | --- | --- | --- | --- | --- | --- | --- |
| **Region part** |  |  |  |  |  |  | **Female** | **PCR (COX1)** | **PCR**  **(18S rRNA)** | **Haplotype (H)** |
| Northeastern | Roi-et (RET) | Thung Khao Luang | 27-Feb-24 | 1 | 16°00'28.1"N 103°55'41.8"E | 131 | 4 | 1 | 3 | 1(H8) |
|  | Maha Sarakham  (MKM) | Mueang | 29-Feb-24 | 2* | 16°09'08.3"N 103°21'57.6"E | 148 | 1 | 1 | 1 | 1 (H2) |
|  |  |  |  | 3* | 16°08'39.8"N 103°22'40.8"E | 153 | 1 | 1 | 1 | 1 (H33) |
|  |  |  |  | 4* | 16°09'08.3"N 103°21'55.4"E | 148 | 4 | 1 | 2 | 1(H34) |
|  | Khon Kaen (KKN) | Mueang | 3-Jan-2024 | 5 | 16°30'22.0"N 102°44'27.6"E | 186 | 0 | 0 | 0 | 0 |
|  |  |  | 11-Nov-2024 | 6 | 16°28'58.08"N 102°48'13.78"E | 201 | 6 | 1 | 2 | 1 (H1) |
|  |  |  | 5-6-Oct-2024 | 7 | 16°31'52.9"N 102°47'20.3"E | 177 | 0 | 0 | 0 | 0 |
|  |  |  | 6-Oct-2024 | 8 | 16°31'09.9"N 102°47'47.9"E | 177 | 0 | 0 | 0 | 0 |
|  | Nong Khai (NKI) | Mueang | 28-Mar-2024 | 9 | 17°51'06.8"N 102°42'39.6"E | 169 | 1 | 1 | 1 | 1(H32) |
|  |  |  |  | 10* | 17°51'41.0"N 102°41'42.0"E | 172 | 8 | 1 | 3 | 1 (H15) |
|  | Bueng Kan (BKN) | Mueang | 29-Mar-2024 | 11 | 18°17'37.7"N 103°34'48.1"E | 170 | 3 | 1 | 2 | 1 (H14) |
|  | Nakhon Phanom (NPM) | Pla Pak | 30-Mar-2024 | 12 | 17°13'18.8"N 104°28'33.6"E | 178 | 2 | 1 | 1 | 1(H11) |
|  | Sakon Nakhon (SNK) | Mueang | 31-Mar-2024 | 13 | 17°06'02.2"N 104°14'20.4"E | 166 | 7 | 1 | 4 | 1(H16) |
|  |  |  |  | 14 | 17°06'11.5"N 104°14'24.0"E | 172 | 1 | 1 | 1 | 1(H27) |
|  | Mukdahan (MDH) | Mueang | 1-April-2024 | 15* | 16°30'49.7"N 104°40'51.6"E | 153 | 7 | 1 | 3 | 1(H8) |
|  | Loei (LEI) | Tha Li | 13-Oct-2024 | 16 | 17°38'20.6"N 101°25'48.9"E | 257 | 0 | 0 | 0 | 0 |
|  |  |  |  | 17 | 17°44'08.5"N 101°26'58.3"E | 236 | 8 | 1 | 4 | 1(H36) |
|  |  |  |  | 18 | 17°41'12.3"N 101°26'06.4"E | 247 | 21 | 2 | 5 | 2(H21,22) |
|  | Chaiyaphum (CPM) | Phu Khiao | 12-Nov-2024 | 19* | 16°28'07.0"N 102°04'45.3"E | 228 | 30 | 1 | 9 | 1(H8) |
|  |  |  |  | 20 | 16°27'37.6"N 102°04'15.0"E | 229 | 2 | 1 | 2 | 1(H20) |
|  | Nakhon Ratchasima (NMA) | Phra Thong Kham | 12-Nov-2024 | 21 | 15°23'16.5"N 102°00'49.8"E | 221 | 3 | 1 | 3 | 1(H10) |
|  | Buriram (BRM) | Nang Rong | 15-Nov-2024 | 22 | 14°34'45.4"N 102°47'55.3"E | 193 | 2 | 1 | 2 | 1(H30) |
|  |  |  |  | 23 | 14°33'52.6"N 102°48'07.7"E | 201 | 0 | 0 | 0 | 0 |
|  |  |  |  | 24 | 14°34'36.6"N 102°48'07.8"E | 195 | 4 | 1 | 4 | 1(H23) |
|  | Ubon Ratchathani (UBN) | Sawang Wirawong | 16-Nov-2024 | 25 | 15°12'54.3"N 105°04'17.5"E | 138 | 3 | 1 | 3 | 1(H24) |
|  |  |  |  | 26 | 15°13'07.3"N 105°04'30.5"E | 137 | 0 | 0 | 0 | 0 |
|  |  |  |  | 27 | 15°12'54.1"N 105°04'45.1"E | 136 | 1 | 1 | 1 | 1(H29) |
| North | Pare (PRE) | Mueang | 8-Aug-2024 | 28 | 18°14'51.1"N 100°08'08.2"E | 184 | 0 | 0 | 0 | 0 |
|  |  |  |  | 29 | 18°14'51.1"N 100°08'08.2"E | 184 | 1 | 1 | 1 | 1(H17) |
|  | Chiang Mai (CMI) | San Kamphaeng | 9-Aug-2024 | 30 | 18°42'45.4"N 99°07'29.4"E | 309 | 15 | 5 | 4 | 5(H3,6,7,8,9) |
|  |  |  |  | 31 | 18°44'27.5"N 99°10'30.8"E | 332 | 0 | 0 | 0 | 0 |
|  |  |  |  | 32 | 18°44'19.8"N 99°10'30.1"E | 334 | 3 | 2 | 2 | 2(H4,5) |
|  |  | Samoeng | 10-Aug-2024 | 33 | 18°54'38.4"N 98°35'31.0"E | 646 | 7 | 1 | 1 | 1(H10) |
|  | Mae Hong Son  (MSN) | Mae Sariang | 13/8/2024 | 34 | 18°12'07.6"N 97°54'49.9"E | 248 | 6 | 1 | 4 | 1(H19) |
|  |  |  |  | 35 | 18°15'10.7"N 97°54'08.7"E | 285 | 0 | 0 | 0 | 0 |
|  |  |  |  | 36 | 18°13'19.3"N 97°54'30.6"E | 261 | 3 | 1 | 2 | 1(H26) |
| Central | Nakhon Sawan (NSN) | Phaisali | 5-Aug-2024 | 37 | 15°32'06.1"N 100°40'50.9"E | 59 | 10 | 1 | 5 | 1(H11) |
|  | Singburi (SBR) | In Buri | 6-Aug-2024 | 38 | 15°02'29.5"N 100°17'07.2"E | 10 | 1 | 1 | 1 | 1(H37) |
|  |  |  |  | 39 | 15°01'58.6"N 100°16'29.5"E | 11 | 7 | 2 | 5 | 2(H17,18) |
|  |  |  |  | 40 | 15°02'27.8"N 100°17'06.4"E | 6 | 0 | 0 | 0 | 0 |
|  |  |  |  | 41 | - | - | 9 | 1 | 2 | 1(H35) |
|  | Phetchabun (PNB) | Chon Daen | 7-Aug-2024 | 42 | 16°09'35.1"N 100°52'49.1"E | 123 | 5 | 1 | 5 | 1(H13) |
|  |  |  |  | 43 | 16°09'47.3"N 100°52'52.3"E | 117 | 2 | 1 | 1 | 1(H11) |
|  |  |  |  | 44 | 16°09'54.7"N 100°52'26.8"E | 111 | 2 | 1 | 1 | 1(H28) |
| Eastern | Chachoengsao (CCO) | Bang Nam Priao | 13-Nov- 2024 | 45 | 13°49'24.0"N 101°00'14.4"E | 3 | 3 | 1 | 2 | 1(H31) |
|  |  |  |  | 46 | 13°49'20.9"N 101°00'25.2"E | 3 | 2 | 1 | 2 | 1(H8) |
|  | Chon Buri (CBRI) | Ko Chan | 13-Nov-2024 | 47 | 13°26'01.5"N 101°19'21.2"E | 39 | 0 | 0 | 0 | 0 |
|  |  |  |  | 48 | 13°26'01.5"N 101°19'21.2"E | 39 | 0 | 0 | 0 | 0 |
|  |  |  |  | 49 | 13°25'12.7"N 101°16'55.8"E | 32 | 0 | 0 | 0 | 0 |
|  | Sa Kaeo (SKW) | Khok Sung | 14-Nov-2024 | 50 | 13°50'32.3"N 102°37'27.1"E | 62 | 0 | 0 | 0 | 0 |
|  |  |  |  | 51 | 13°50'43.0"N 102°38'25.6"E | 57 | 0 | 0 | 0 | 0 |
|  |  |  |  | 52 | 13°50'27.8"N 102°39'11.1"E | 58 | 0 | 0 | 0 | 0 |
|  |  |  |  | 53 | 13°50'30.1"N 102°39'05.7"E | 56 | 0 | 0 | 0 | 0 |
| Western | Kanchanaburi (KRI) | Thong Pha Phum | 11-Jun-2024 | 54 | 14°21'07.8"N 98°57'28.7"E | 246 | 0 | 0 | 0 | 0 |
|  |  |  |  | 55 | 14°34'24.2"N 98°45'02.2"E | 78 | 1 | 1 | 1 | 1(H12) |
|  | Phetchaburi (PBI) | Cha-am | 5-Jun-2024 | 56 | 12°38'27.9"N 99°51'54.5"E | 60 | 4 | 2 | 3 | 2(H3,20) |
| Southern | Chumphon  (CPN) | Pathio | 6-Jun-2024 | 57 | 10°55'05.5"N 99°26'39.1"E | 25 | 0 | 0 | 0 | 0 |
|  |  |  |  | 58 | 10°51'50.2"N 99°23'44.7"E | 12 | 0 | 0 | 0 | 0 |
|  |  |  |  | 59 | 10°47'58.0"N 99°22'38.0"E | 96 | 0 | 0 | 0 | 0 |
|  | Nakhon Si Thammarat (NST) | Tha Sala | 8-Jun-2024 | 60 | 8°38'24.4"N 99°54'14.0"E | 4 | 1 | 1 | 1 | 1(H25) |
|  |  |  |  | 61 | 8°38'13.3"N 99°53'56.5"E | 8 | 0 | 0 | 0 | 0 |
|  |  |  |  | 62 | 8°38'13.5"N 99°54'52.1"E | 10 | 0 | 0 | 0 | 0 |
| Total | 26 | 27 | - | 62 | - | - | 201 | 47 | 100 | 37 |

(*) Hand collection was employed for these specimens because they did not enter the traps

**Table A.2** Climatic conditions at sampling sites during the collection period,

including temperature, humidity, and monthly average rainfall by province.

| **No. Farm** | **Temperature Primary data (°C) *** | **Monthly mean temperature (°C) **** | **Monthly mean humidity (°C) **** | **Rainfall (mm) **** | **Number of cattle and buffalo** |
| --- | --- | --- | --- | --- | --- |
| 1 | 36 | 28.85 ± 2.48 | 68 | - | 4 |
| 2 | 35 | 28.4 ± 2.38 | 62.4 | 0.4 | 10 |
| 3 | 35 | 28.4 ± 2.38 | 62.4 | 0.4 | 6 |
| 4 | 35 | 28.4 ± 2.38 | 62.4 | 0.4 | 4 |
| 5 | - | 28.4 ± 2.38 | 61.6 | 0.4 | 4 |
| 6 | 32.5 | 25.19 ± 1.64 | 68.3 | - | 10 |
| 7 | - | 26.66 ± 1.65 | 76.9 | - | 8 |
| 8 | - | 28.09 ± 1.65 | 76.9 | 27.7 | 4 |
| 9 | 33.9 | 28.09 ± 0.76 | 68.5 | 27.7 | 2 |
| 10 | 33.9 | 28.85 ± 2.28 | 68.5 | 28.9 | 12 |
| 11 | 35 | 28.85 ± 2.28 | 73.2 | 28.9 | 16 |
| 12 | 28.4 | 27.2 ± 2.08 | 65 | 28.9 | 10 |
| 13 | 35.1 | 28.41 ± 2.36 | 66.3 | 28.4 | 10 |
| 14 | 36.4 | 28.44 ± 2.29 | 66.3 | 28.2 | 7 |
| 15 | 38.1 | 28.44 ± 2.29 | 65 | 28.2 | 14 |
| 16 | 42.5 | 28.29 ± 2.18 | 81.3 | 29.1 | 8 |
| 17 | 37.4 | 26.99 ± 0.8 | 81.3 | 67.6 | 7 |
| 18 | 28.8 | 26.99 ± 0.8 | 81.3 | 67.6 | 21 |
| 19 | 32.2 | 27.34 ± 1.39 | 65.4 | 67.6 | 6 |
| 20 | 34.6 | 27.34 ± 1.39 | 65.4 | 10.3 | 5 |
| 21 | 30.5 | 27.49 ± 1.52 | 71 | 10.3 | 7 |
| 22 | 29 | 26.62 ± 1.43 | 73 | 10.6 | 3 |
| 23 | 26.2 | 26.62 ± 1.43 | 73 | 7.4 | 20 |
| 24 | 26.2 | 26.62 ± 1.43 | 73 | 7.4 | 14 |
| 25 | 31.8 | 27.08 ± 1.94 | 69 | 7.4 | 9 |
| 26 | 31.1 | 27.08 ± 1.94 | 69 | 0.5 | 7 |
| 27 | 27.2 | 27.08 ± 1.94 | 69 | 0.5 | 15 |
| 28 | 32.8 | 28.57 ± 1.2 | 86 | 0.5 | 20 |
| 29 | 33.5 | 28.57 ± 1.2 | 86 | 298.5 | 10 |
| 30 | 44.1 | 29.07 ± 1.19 | 81 | 298.5 | 20 |
| 31 | - | 29.07 ± 1.19 | 81 | 237.7 | 8 |
| 32 | 33 | 29.07 ± 1.19 | 81 | 237.7 | 4 |
| 33 | 29.8 | 27.73 ± 1.15 | 96 | 237.7 | 7 |
| 34 | 28.9 | 28.09 ± 0.93 | 86.3 | 237.7 | 9 |
| 35 | 29.4 | 28.09 ± 0.93 | 86.3 | 385.9 | 7 |
| 36 | 26.9 | 28.09 ± 0.93 | 86.3 | 385.9 | 15 |
| 37 | 39 | 29.43 ± 1.1 | 80 | 385.9 | 19 |
| 38 | 30 | 29.98 ± 1.1 | 81 | 254.4 | 11 |
| 39 | 30.9 | 29.98 ± 1.1 | 81 | 404.6 | 11 |
| 40 | - | 29.98 ± 1.1 | 81 | 404.6 | 12 |
| 41 | 36.4 | 29.98 ± 1.1 | 81 | 404.6 | 6 |
| 42 | 35.3 | 29.21 ± 1.25 | 81 | 404.6 | 27 |
| 43 | 36 | 29.21 ± 1.25 | 81 | 42.3 | 8 |
| 44 | 28 | 29.21 ± 1.25 | 81 | 42.3 | 9 |
| 45 | 32.2 | 29.77 ± 0.82 | 79 | 42.3 | 6 |
| 46 | 40.3 | 29.77 ± 0.82 | 79 | 5.2 | 5 |
| 47 | - | 29.56 ± 0.83 | 74 | 5.2 | 2 |
| 48 | - | 29.56 ± 0.83 | 74 | 73.5 | 13 |
| 49 | - | 29.56 ± 0.83 | 74 | 73.5 | 2 |
| 50 | - | 28.65 ± 1.22 | 71 | 73.5 | 8 |
| 51 | - | 28.65 ± 1.22 | 71 | 56.7 | 5 |
| 52 | - | 28.65 ± 1.22 | 71 | 56.7 | 8 |
| 53 | - | 28.65 ± 1.22 | 71 | 56.7 | 3 |
| 54 | - | 28.74 ± 0.96 | 86 | 56.7 | 200 |
| 55 | 26.8 | 28.74 ± 0.96 | 86 | 295.1 | 7 |
| 56 | 26.5 | 29.53 ± 0.63 | 71 | 295.1 | 56 |
| 57 | - | 29.68 ± 1.56 | 78 | 91.7 | 12 |
| 58 | - | 29.68 ± 1.56 | 78 | 91.7 | 21 |
| 59 | - | 29.68 ± 1.56 | 78 | 91.7 | 10 |
| 60 | 27.1 | 29.81 ± 0.78 | 78 | 91.7 | 15 |
| 61 | - | 29.81 ± 0.78 | 79 | 169.8 | 4 |
| 62 | - | 29.81 ± 0.78 | 79 | 169.8 | 27 |

* Temperature was measured directly in the field and recorded as primary data. ** Secondary data (Source: Monthly temperature and rainfall data partially obtained from the Thai Meteorological Department (2025)).

**Table A.3** Haplotype diversity (Hd) and percentage sequence similarity of *Tabanus rubidus* COI sequences (624 bp) from beef-cattle farming areas in Thailand, compared with reference sequences from GenBank.

| **Haplotype** | **Code of *T. rubidus* in this study** | **GenBank accession number** | **% Similarity** | **Country** |
| --- | --- | --- | --- | --- |
| H1 | KKN2 | MG426146 | 99.68% | Thailand |
| H2 | MKM2 | MG426144 | 100.00% | Thailand |
| H3 | PBI18, CMI30 | MG426150 | 100.00% | Thailand |
| H4 | CMI1 | MG426146 | 99.52% | Thailand |
| H5 | CMI2 | MG426144 | 99.84% | Thailand |
| H6 | CMI8 | MG426153 | 99.68% | Thailand |
| H7 | CMI11 | MG426142 | 99.36% | Thailand |
| H8 | CMI24, RET1, MDH1, CCO22, CPM31 | MG426146 | 99.84% | Thailand |
| H9 | CMI35 | MG426146 | 99.52% | Thailand |
| H10 | CMI77 | MG426151 | 99.36% |  |
| H11 | NSN8, PNB38, NPM6, NMA6 | MG426146 | 100.00% | Thailand |
| H12 | KAI11 | MG426146 | 99.36% | Thailand |
| H13 | PNB8 | MG426146 | 98.88% | Thailand |
| H14 | BKN1 | MG426146 | 98.88% | Thailand |
| H15 | NKI2 | MG426146 | 99.68% | Thailand |
| H16 | SNK5 | MG426143 | 98.72% | Thailand |
| H17 | PRE12, SBR3 | MG426146 | 99.84% | Thailand |
| H18 | SBR11 | [MG426144](https://www.ncbi.nlm.nih.gov/nucleotide/MG426144.1?report=genbank&log$=nucltop&blast_rank=1&RID=PRHV93KD016) | 99.84% | Thailand |
| H19 | MSN3 | MG426146 | 99.04% | Thailand |
| H20 | PBI8, CPM33 | MG426146 | 98.72% | Thailand |
| H21 | LEI24 | MG426146 | 99.20% | Thailand |
| H22 | LEI42 | MG426146 | 99.68% | Thailand |
| H23 | BRM31 | MG426146 | 99.52% | Thailand |
| H24 | UBN2 | MG426146 | 99.68% | Thailand |
| H25 | NST8 | MZ769389 | 99.84% | Malaysia |
| H26 | MSN5 | MG426147 | 99.84% | Thailand |
| H27 | SNK22 | MG426146 | 99.84% | Thailand |
| H28 | PNB31 | MG426146 | 99.52% | Thailand |
| H29 | UBN66 | MG426146 | 99.52% | Thailand |
| H30 | BRM8 | MG426142 | 99.68% | Thailand |
| H31 | CCO19 | MG426153 | 99.68% | Thailand |
| H32 | NKI1 | MG426153 | 98.40% | Thailand |
| H33 | MKM3 | MG426146 | 99.36% | Thailand |
| H34 | MKM6 | MG426146 | 99.52% | Thailand |
| H35 | SBR30 | MG426150 | 99.68% | Thailand |
| H36 | LEI06 | MG426146 | 99.84% | Thailand |
| H37 | SBR65 | MG426147 | 99.84% | Thailand |

**Table A.4** List of Tabanidae COI sequences used for median-joining network analysis, showing geographical origins and database identifiers.

| **Source / Origin** | **Country** | **No. of Sequences** | **Accession Numbers / BOLD Process IDs** |
| --- | --- | --- | --- |
| This Study | Thailand | 47 | V065691-PV065737 |
| BOLD System | Thailand | 31 | THAMA1005-22,  HAMA1006-22,  THAMA3388-22, THAMA3389-22, THAMA953-22, THAMA954-22, THAMA955-22, THAME028-22, THAME029-22, THAME085-22, THAME086-22, THAME16665-23, THAME9303-23, THAMF12353-23, THAMF2477-23, THAMF2478-23, THAMF2479-23, THAMF2480-23, THAMF2481-23, THAMF2482-23, THAMF2483-23, THAMG2807-23, THAMG2808-23, THAMG2813-23, THAMG6985-23, THAMG6986-23, THAMG6987-23, THAMG6988-23, THAMG6989-23, THAMG6990-23, THAMG6993-23 |
| GenBank (NCBI) | Thailand | 11 | MG426142, MG426143, MG426144, MG426145, MG426146, MG426147, MG426148, MG426149, MG426150, MG426151, MG426153 |
| GenBank (NCBI) | India | 6 | KM111704, KM111663, KM111705, KM111714, KM111705, KM111662 |
| GenBank (NCBI) | Malaysia | 1 | MZ769389 |
| BOLD System | Pakistan | 1 | MADIP405-11 |
| BOLD System | Bangladesh | 10 | BIOUG23285-C11  GMBCD3148-15  GMBCE2134-15  GMBCE2135-15  GMBCF3466-15  GMBCF3467-15 GMBCF3469-15  GMBCF3472-15  GMBCH3841-15  GMBDB1938-23 |
| **Total** |  | **107** |  |
